# Associations of Dietary and Lifestyle Components with Atrial Fibrillation

**DOI:** 10.3390/nu16030456

**Published:** 2024-02-05

**Authors:** Klaudia Leszto, Weronika Frąk, Szymon Kurciński, Julia Sinkowska, Sylwia Skwira, Ewelina Młynarska, Jacek Rysz, Beata Franczyk

**Affiliations:** 1 Department of Nephrocardiology, Medical University of Lodz, Ul. Zeromskiego 113, 90-549 Lodz, Poland; claudial@op.pl (K.L.); julia.sinkowska@stud.umed.lodz.pl (J.S.); sylwia.skwira@stud.umed.lodz.pl (S.S.);; 2 Department of Nephrology, Hypertension and Family Medicine, Medical University of Lodz, Ul. Zeromskiego 113, 90-549 Lodz, Poland

**Keywords:** atrial fibrillation, risk factors, non-pharmacological therapies, physical activity, obesity, weight loss, diet, sleep, smoking, sedentary lifestyle

## Abstract

Atrial fibrillation (AF) is a prevalent cardiac arrhythmia that still remains a significant health concern, especially due to its consequences, including stroke and heart failure. This review explores the intricate interplay between AF, lifestyle choices, and dietary habits. It is particularly focused on findings from diverse studies about non-pharmacological methods of managing AF. Moreover, its purpose is to elucidate the implementation of lifestyle changes such as physical activity or proper diet choices in the integrated treatment strategy of patients with AF.

## 1. Introduction

Atrial fibrillation (AF) is a supraventricular tachyarrhythmia characterized by uncoordinated atrial electrical activation and, consequently, ineffective atrial contraction, in accordance with the 2020 Guidelines for Management of Atrial Fibrillation by the European Society of Cardiology (ESC). The electrocardiographic features of AF include irregularly irregular R-R intervals (when atrioventricular conduction is not impaired), the absence of distinct repeating P waves, and irregular atrial activations [1,2]. AF is the most frequent arrhythmia managed in clinical practice, and it is associated with an elevated risk of mortality, stroke, and peripheral embolism [2].

The epidemiology of AF reveals a rising global incidence and prevalence [3]. Its prevalence is estimated to affect up to 37.574 million individuals within the general population [4]. Studies have shown that old age, male gender, and European descent are important risk factors for developing AF. Other modifiable risk factors include a sedentary lifestyle, smoking, obesity, diabetes mellitus, obstructive sleep apnea, and high blood pressure predisposing to AF, and each has been shown to induce structural and electrical atrial remodeling. Both heart failure and myocardial infarction increase the risk of developing AF and vice versa, creating feedback that increases mortality [5,6,7,8].

AF can be classified into several types based on the presentation, duration, and spontaneous termination of AF episodes. Paroxysmal AF is characterized by irregular heartbeats that come and go spontaneously, with episodes lasting minutes to hours. Persistent AF involves irregular heart rhythms lasting more than seven days, often requiring medical intervention or electrical cardioversion. Further, long-standing persistent AF is recognized when AF persists for more than a year. When restoring a normal heart rhythm is unsuccessful or not pursued, it is a permanent AF [1,9,10].

Symptoms of AF differ in severity and include palpitations, fatigue, shortness of breath, dizziness, and, less commonly, chest pain or tightness. The diverse clinical presentation makes early detection and personalized treatment crucial [11,12,13].

Managing AF involves a multifaceted approach, considering the underlying causes, symptom severity, and associated risk factors. Treatment strategies are broadly categorized into rhythm control and rate control measures. Rhythm control may involve medications like amiodarone or flecainide and cardioversion procedures. Rate control utilizes medications such as beta-blockers and calcium channel blockers to slow down the heart rate [1,14,15,16]. Anticoagulant medications, such as warfarin or direct oral anticoagulants (DOACs), are often prescribed to reduce the risk of blood clot formation and stroke in individuals with AF [1,17,18]. It is worth mentioning “the simple Atrial fibrillation Better Care (ABC) holistic pathway”, which comprises the following: ‘A’ Anticoagulation/Avoid stroke; ‘B’ Better symptom management; and ‘C’ Cardiovascular and Comorbidity optimization, consistent with ESC guidelines for AF. Coordinated care for patients with AF is associated with reduced risks across several critical parameters: all-cause mortality, a decreased occurrence of the composite outcome involving stroke, major bleeding, and cardiovascular death, a reduced incidence of the first hospitalization, as well as diminished rates of cardiovascular events and lower healthcare-related costs [1].

The purpose of this paper is to present the recent and on-going non-pharmacological methods that can be implemented in the AF patient group. A growing body of evidence has implicated the use of non-pharmacological strategies in the therapy of AF. We summarized physical activity together with dietary and lifestyle adjustment, in particular physical activity, the influence of smoking tobacco and alcohol consumption, the role of sleep, and dietary management. A holistic approach encompassing a heart-healthy diet, regular physical activity, and attention to nutritional status is integral to the comprehensive care of individuals with AF. These lifestyle modifications not only aid in symptom management but also contribute to slowing the development and progression of AF. Hence, we believe that relevant knowledge of these topics might be helpful in the management of this disease, which is significant for proper medical therapy.

## 2. Lifestyle

An unhealthy lifestyle is one of the risk factors for AF [1]. Lifestyle changes have been described as the “fourth pillar” of AF management, added to rhythm control, rate control, and anticoagulation [19]. In this chapter, we focused on physical activity, smoking, and quality of sleep.

### 2.1. Physical Activity

Physical activity reduces the risk of AF in two ways. Firstly, it helps to control other modifiable risk factors such as atrial hypertension or being overweight [19,20]. Secondly, studies have reported that physical activity independently decreases the chance of AF [21].

Decreasing AF risk was noted in people who reported regular physical activity [21,22,23,24]. MET (metabolic equivalent task) is a method for calculating the energy cost of physical activity in the form of a multiple of the resting metabolic rate [25]. At least 500 MET-min/wk (metabolic equivalent task min per week) of regular exercise is currently recommended [26]. This amount of physical activity reduces the incidence of AF to a greater extent amongst females than males. The guideline-recommended physical activity also reduces the risk of ventricular arrhythmia incidents but has no effect on the occurrence of bradyarrhythmias [27].

Moo-Nyun Jin et al., in the cohort study, divided the general Korean population into four groups: (1) inactive group: 0 MET-min/wk, (2) insufficiently active group: 1–500 MET-min/wk, (3) active group: 500–1000 MET-min/wk, and (4) highly active group: >1000 MET-min/wk. In the active group, the risk of AF was reduced by 12% compared to the inactive group. The risk decreased, respectively, by 6% and 7% in insufficiently active and highly active groups [28].

Differences between studies may arise from diverse research groups and the method of physical activity assessment (self-reporting). However, researchers agree that moderate-intensity physical activity reduces the frequency of AF incidents [27,28,29]. Some studies reported that high activity in females could be beneficial for AF prevention, as opposed to the male population [28,30,31].

Physical activity plays a significant role not only in primary prevention but also in secondary prevention and treatment of AF. Improvements in quality of life and symptoms of AF were reported in patients with primarily permanent AF as a result of physical exercise [32,33,34]. Ischemic stroke is one of the most serious complications of AF. Herber et al. suggest the neuroprotective effects of physical activity in patients with AF. The research shows that patients concerned with physical activity presented a lower frequency of ischemic brain infarcts, white matter disease, and cerebral microbleeds. They also had a larger normalized brain volume and better results on cognitive tests. The authors recommend that patients with AF should be encouraged to be physically active in order to prevent complications from the nervous system [35].

An important question is as follows: what type of exercise is safe and most beneficial for patients with AF? Positive results of cardiac rehabilitation were confirmed in the case of moderate-to-vigorous intensity continuous training (MICT) in patients with persistent or permanent AF. Patients reported improvements in quality of life, reduction in symptoms, better functional capacity, and cardiorespiratory fitness [36,37]. An example of MICT as cardiac rehabilitation may be a set of exercises in Table 1 (in one-hour sessions, twice a week) [38]. High-intensity interval training (HIIT) was also identified as profitable. Reed et al. compared the results of MICT and HIIT-based rehabilitation in patients with persistent and permanent AF. Both have improved functional capacity (measured in a six-minute walk test), quality of life (in aspects such as physical and social functioning, bodily pain, and vitality), and a decrease in BP and HR. Also, both methods were safe for patients’ health. The short duration of HIIT may be an advantage for patients who reported neglect of cardiac rehabilitation due to a lack of time [37].

Examination of aerobic interval training (AIT) for 12 weeks in patients with paroxysmal or persistent AF showed a decline of 3.3% in the time in AF (from 8.1% to 4.8%), a decrease in AF symptoms, a reduction in resting HR, and an improvement in vitality and general health. In the AIT group, there was also a smaller number of hospital admissions and cardioversions. The study reported that AIT training as a form of cardiac rehabilitation in patients with AF is safe, and no major adverse outcomes were seen [39]. The types of training, together with the outcomes of rehabilitation with various training methods, are summarized accordingly in Table 1 and Table 2.

On the other hand, physical activity can be not only a prevention but also a trigger factor for AF. Endurance exercises cause structural, functional, and electrophysiological alterations [40]. Premature atrial contractions are more frequent in professional athletes [41]. They may trigger AF occurrence [42] and lead to permanent AF [43]. Other examples of lesions associated with AF in endurance athletes are as follows: increased vagal tone [44], enlargement of the left atrium, and pulmonary vein ectopy [42]. These factors have been confirmed by Guash et al. in the animal experiment (using rats), simulating endurance training [45]. Moreover, similar findings were demonstrated by Aizer et al. in their research on humans [46].

Petrungaro et al. indicate that the mechanism of AF in elite athletes is still largely speculative [47]. A large number of studies have shown the higher frequency of this arrhythmia, for example, in marathon runners [46], runners exercising 7 days per week, and long-term competitive swimmers [48]. However, some theses question these results and raise doubts in the scientific community. Clarke et al. identified a progressive increase in survival rates among Olympic athletes, especially in endurance sports [49]. Marijon et al. observed 40% less mortality in the Tour de France participants than in the general population [50]. Currently, it is not possible to clearly establish a correlation between physical activity and AF occurrence. Recent studies indicate the great role of sex in this association. High amounts of vigorous physical activity led to a 12% increase in AF incidents in males, but in females, vigorous physical activity was related to an 8–16% reduction [27]. Another piece of evidence to prove that the association between sport and physical activity is sex-linked is a meta-analysis of 23 cohort studies by Kunustor et al. It was confirmed that regular physical activity could be associated with an increased risk of AF in men and a decreased risk in women [51]. All these issues require further research, especially those related to the possible confounding factors in the cohort of professional athletes. Potential misleading factors include a lower incidence of other risk factors such as smoking or obesity, a reduced chance for asymptomatic AF (because of regular and intense physical activity), frequent and detailed medical examinations, and the use of illicit drugs, which may be a trigger factor for arrhythmia [47]. To sum up, it is justified to suppose that endurance sports may promote the occurrence of AF, especially in males. Further research is needed to evaluate this thesis and upgrade guidelines in sports medicine in order to improve the safety of professional sports athletes.

### 2.2. Smoking Tobacco

Tobacco smoking is the leading avoidable cause of death in the world and a very significant risk factor for cardiovascular disease. Smoking has been recognized as a very severe public health problem, but still, it is considered socially acceptable compared to, for example, drugs and narcotics. Cigarette smoke contains more than 7000 chemicals, such as nicotine, carbon monoxide, tar, and many others [52,53]. Smoking is a major risk factor for AF, and there is a dose-related manner between pack years and the increased danger of AF [54].

Nicotine contained in cigarettes has a direct impact on ion channel conduction in atrial myocytes. It can cause a delay in ventricular repolarization and also lengthen the effective refractory period by blocking outward K+ current [55].

Tobacco smoking contributes to increased atrial fibrosis, which favors the event of atrial arrhythmias by slowing down the conduction of electrical impulses in cardiac tissues. It was shown in the canine model that atrial fibrosis is also caused because nicotine induces downregulation of microRNAs in atrial fibroblasts and upregulation of transforming growth factors, which leads to higher production of collagen [56,57].

Nicotine also has an impact on sympathetic neurotransmission. A higher concentration of catecholamine in plasma [58] leads to increased blood pressure [59], a resting heart rate, and a greater risk of hypertension [60,61,62]. All of that is connected with a bigger risk of AF [63,64].

People with AF are at high risk for stroke and thromboembolism. There is a special score for AF stroke risk—CHA2DS2-VASc. It is very helpful for defining stroke risk among affected people [65]. Anticoagulation therapy is usually initiated when the CHA2DS2-VASc score is ≥2. This score is higher among people who smoke than among nonsmokers. Therefore, the risk of thromboembolic stroke is much higher for smokers with AF [66].

Heat-not-burn tobacco products use batteries to heat tobacco to a maximum of 350 degrees, which is much lower than the temperature used for heating conventional cigarettes. A lot of people think that those heated tobacco products are less harmful. However, aerosols of heat-not-burn products contain the same chemicals as conventional cigarette smoke: acrolein, formaldehyde, benzaldehyde, acenaphthylene, nicotine, and carbon monoxide. Changing the method of delivery of these substances does not reduce their harmfulness [67].

The Rotterdam Study showed that current and former smokers had a higher risk of AF in comparison to people who had never smoked. There are no differences between women and men [54]. Even early exposure to secondhand smoke throughout gestational development and childhood might heighten the risk of AF later in life, even by 37% [68]. The best strategy to avoid AF is to never start smoking.

### 2.3. Sleep

The most common sleep-related breathing disorder is obstructive sleep apnea (OSA). It is characterized by recurring apneas and hypopneas caused by complete or partial collapse of the upper airway during sleep. An episode of hypopnea should meet one of the following criteria: (1) >50% decrease in the flow of air or tidal volume for 10 s or more, (2) moderate lowering in airflow (<50%) with >3% arterial oxygen desaturation, (3) moderate reduction in the flow of air with proof of awakening from sleep in the electroencephalogram. A polisomnographic study is a gold standard for the diagnosis of obstructive sleep apnea; it records sleep and breathing overnight in a sleep laboratory [69].

In population-based studies, the prevalence of OSA ranges from 3 to 49%, and among patients with AF, it ranges from 21 to 74% [70]. The Sleep Heart Health Study found that the risk of AF among patients with OSA is four times higher than that without OSA.

There are multiple mechanisms underlying AF that are attributable to OSA. Those recurrent cessations in ventilation caused by upper airway collapse result in hypoxia, intrathoracic pressure shifts, cardiac autonomic nervous system hyperactivity, systemic inflammation, and oxidative stress [71,72].

#### 2.3.1. Hypoxia

OSA leads to recurrent episodes of hypoxia that cause sympathetic nerve activity and chemoreflex, which result in higher blood pressure and tachycardia, particularly at the end of the episodes of apnea [73]. Myocardial oxygen demand is heightened because of tachycardia and high blood pressure, but myocardial oxygen supply is very low due to hypoxia. A consequence of that is promoting AF because of repeated myocardial and, therefore, atrial ischemia while sleeping.

#### 2.3.2. Intrathoracic Pressure Changes

Upper airway occlusion generates a hasty drop in intrathoracic pressure (e.g., −65 mm Hg). It is caused by ineffective respiratory efforts against an obstructed airway. These large oscillations of pressure cause increased left ventricular afterload and compromise the thin-walled atria, leading to their acute distension. These changes in the ventricle and atria may activate the stretch-mediated ion channels and cause cardiac remodeling, hence enhancing the tendency to arrhythmia. These episodes of obstructive apnea lead to corrected QT interval prolongation and progressive increases in atrial premature beat frequency, which have been known as precursors to AF [74,75].

Swings in intrathoracic pressure during obstructive apneas can cause cardiac wall stress and atrial stretch, which may lead to fibrosis at anchoring atrial regions, for example, the pulmonary vein ostium, a site that is critical to AF induction [76,77,78].

#### 2.3.3. Cardiac Autonomic Nervous System Hyperactivity

Cardiac electrical function is altered by the interplay between the parasympathetic and sympathetic arms of the autonomic nervous system. Among patients with OSA, there are sudden changes in cardiac autonomic balance that are dependent on the apneic episode phases. During apnea, there is significant bradycardia, which means parasympathetic dominance. Right after the apnea, there is a strong rebound tachycardia, which is likely due to increasing cardiac sympathetic activity. After that, the heart rate gradually returns to the resting level [79]. After the apnea, arterial blood pressure also rises, suggesting vascular sympathetic activity [80]. These sequential autonomic modifications lead to enhanced susceptibility to arrhythmia.

#### 2.3.4. Systemic Inflammation and Oxydative Stress

Cycles of hypoxia and reoxygenation in OSA cause activation of a proinflammatory cascade that drives ROS formation and nitric oxide reduction that may result in an enhanced state of systemic inflammation and oxidative stress [81,82]. This action leads to endothelial damage and an increased propensity for arrhythmias [83]. For example, C-reactive protein and IL-6 are systemic inflammatory markers. These two markers are elevated among patients with OSA, and both of them are associated with endothelial dysfunction and dilatation of the left atrium, which are known AF contributors [84,85].

OSA is the most well-studied and confirmed risk factor for AF connected with sleep. However, other types of sleep disturbances may also be related to the occurrence of AF [86]. Christensen et al. reported that frequent awakening at night and longer sleep onset latency are associated with prevalent AF. Self-reported night-time awakening was connected with a 33% higher risk of AF. Moreover, pre-existing insomnia affects the 36% growth in the chance of incident AF. These results were elaborated after adjusting for potential confounders and evidence of OSA. The obtained results indicate that the risk connected with insomnia is at a similar level as in smoking and OSA. Shortening REM sleep may also increase the risk of AF [87]. Genuardi et al. reported that each 1 h sleep reduction correlates with a 17% greater risk of prevalent AF and a 9% greater risk of incident AF [88]. It is, therefore, likely that quality and duration of sleep are relevant in the pathogenesis and occurrence of AF, and they may become the aim of prevention and treatment [87,88]. Moreover, patients with AF with severe OSA show a lower response rate to antiarrhythmic treatment than those with milder OSA [89]. Kanagala and colleagues showed that patients with OSA have a greater recurrence rate after initially successful cardioversion than patients without OSA [90]. The atrial fibrillation recurrence rate after pulmonary vein isolation (PVI) is 31% higher among patients with OSA [91,92]. This means that sleep apnea reduces the effectiveness of AF treatment. Additionally, the VARIOSA-AF (Variability of Sleep Apnea Severity and Risk of Atrial Fibrillation) study showed that most patients with AF do not have severe OSA every day but rather mild or moderate sleep apnea and considerable intraindividual night-to-night variability. Linz et al. [93] described in a rat model that oxidative stress during mild or moderate OSA is completely reversible within 24 h, but 3 weeks of repeated exposure to oxidative stress, even every second day, result in an arrhythmogenic AF substrate. Although treatment of OSA is mainly recommended for patients with severe OSA and AF, findings from the Linz et al. study suggest that even mild-to-moderate OSA with high night-to-night variability is sufficient to result in an arrhythmogenic substrate. The gold standard for OSA therapy is CPAP. The positive pressure prevents the pharyngeal area from collapsing, and it helps ease airway obstruction [94]. In patients with AF and OSA, CPAP use was related to the maintenance of sinus rhythm. People undergoing cardioversion and receiving CPAP treatment were less likely to have AF recurrence in comparison to untreated people—42% with CPAP vs. 82% without CPAP. The same situation is with patients undergoing PVI; CPAP helps to maintain a lower recurrence rate of AF—28% with CPAP treatment after PVI vs. 63% without CPAP treatment [90,95].

In summary, the impact of OSA treatment on reducing recurrences of AF is very important. It means that close interdisciplinary collaboration between cardiologists, electrophysiologists, and sleep specialists is essential when it comes to the management of OSA in patients with AF.

### 2.4. Air Pollution

Air pollution, as defined by the World Health Organization, includes indoor and outdoor contamination of air by chemical, physical, or biological agents that pose a potential threat to both ecosystems and human health [96]. This makes it a probable risk factor for the development of lifestyle-related diseases. Currently, one area undergoing intensive analysis is the assessment of the impact of air pollutants, like particulate matter (PM), ground-level ozone (O_3_), sulfur dioxide (SO_2_), nitrogen dioxide (NO_2_), and carbon monoxide (CO), on the occurrence and progression of arrhythmias, including, among others, AF [97].

As a consequence of exposure to elevated concentrations of these substances, diverse processes are triggered, lowering the threshold for the onset of AF [97,98]. Among the proarrhythmic mechanisms, alterations primarily involve the progression of systemic inflammation, dysfunction of the autonomic nervous system, and structural remodeling of the myocardium, which lead to the occurrence of electrophysiological changes like heightened excitability of the cell membrane of cardiomyocytes, an increased risk of both early and delayed depolarizations, and modifications in conduction velocity, depolarization, and repolarization [97]. In numerous studies, it has been underscored that both the concentration and duration of exposure to the investigated substance exert an influence on the extent of these electrophysiological alterations, thereby affecting the potential occurrence of AF [97,98,99].

Analyzing prolonged exposure to air pollution, it is noteworthy to cite the findings of the cohort study conducted by Shin et al. This study explored a 15-year exposure to PM2.5 (particulate matter ≤ 2.5 μm in aerodynamic diameter), NO_2_, and O_3_ in a cohort of 5,071,956 individuals aged 35–85 years with previously undiagnosed AF. The results identified 313,157 cases of AF in the studied population, highlighting that even relatively low threshold concentrations of air pollutants (6 µg/m^3^ for PM2.5 and 25 ppb for O_3_) significantly elevate the risk of AF occurrence. Additionally, it is worth noting that individuals diagnosed with AF were more often males and suffered from cardiovascular diseases twice as frequently compared to the general population [99].

Kim et al. studied 432,587 individuals without a history of AF exposed to PM2.5, PM10( ≤10 μm in aerodynamic diameter), and gaseous air pollutants (SO_2_, CO, NO_2_). They made similar observations, reporting 5825 AF incidents within 1,666,528 person-years. The risk of arrhythmia correlated with increases in concentration: PM2.5 (HR = 1.179 for a 10 µg/m^3^ increase, *p* < 0.001), PM10 (HR = 1.034 for a 10 µg/m^3^ increase, *p* < 0.001), SO_2_: HR = 1.008 for a 10 ppb increase, *p* < 0.001, NO_2_: HR = 1.016 for a 10 ppb increase, *p* < 0.001, CO: HR = 1.022 for a 1 ppm increase, *p* < 0.001. Also in this study, a higher risk of AF after chronic exposure to PM2.5 was particularly notable in males (HR-1.187 (1.183–1.192), *p* < 0.001), older individuals (aged ≥ 60 years; HR-1.194 (1.183–1.199), *p* < 0.001), individuals with a body mass index (BMI) ≥ 27.5 kg/m^2^ (HR-1.191 (1.183–1.199), *p* < 0.001), and those with a documented history of arterial hypertension (HR-1.191 (1.185–1.197); *p* < 0.001) [100].

In several studies, it has also been demonstrated that an increase in the risk of atrial fibrillation episodes occurs during short-term exposure to air pollutants [101,102]. Kwon et al. demonstrated that short-term exposure to a 10 µg/m^3^ increase in air pollutant PM2.5 concentration was associated with a 4.5% increase in emergency department visits for AF in a nationwide cohort of the general population in Korea [101]. These findings align with observations made by Hsiu Hao Lee et al. in 2019, who reported that in a cohort of 670 patients from Taiwan, short-term exposure to PM2.5 air pollutants was associated with approximately a 20% increase in hospitalizations for atrial fibrillation in the first 2 days following exposure [102]. Additionally, it is worth mentioning that despite the findings by Kwon et al. indicating that short-term exposure to PM2.5 leads to changes in electrophysiological mechanisms, increasing the risk of AF episodes, the second part of the study analyzing long-term exposure to air pollutants did not show a clear association with the occurrence of AF [101].

Although direct comparison of studies is challenging due to heterogeneous air pollution levels and diverse demographic characteristics of the studied populations, individual analyses regarding air pollution exposure and the incidence of AF consistently point to a new, relevant risk factor for arrhythmias. Considering the increased risk of stroke and overall mortality associated with AF, it is necessary to make further efforts to reduce air pollution exposure worldwide in order to minimize the negative impact on the health of the general population.

## 3. Diet

### 3.1. Dietary Patterns

Diet modification is the foundation of cardiovascular disease prevention. A nutritious diet supports cardiovascular health, helping to manage risk factors such as hypertension, obesity, and high cholesterol, which are leading to AF. Chronic inflammation is a strong risk indicator of AF [103]. A diet rich in anti-inflammatory foods can be beneficial. Moreover, a heart-healthy diet, low in sodium and high in potassium, can contribute to blood pressure control, which is also one of the risk factors for AF. Adopting a balanced diet helps with weight management, reducing the strain on the cardiovascular system. There is a growing body of evidence supporting the role of diet in the treatment of cardiovascular disease and its ensuing complications.

One of the most recommended is the Mediterranean diet, known for its emphasis on fruits, vegetables, whole grains, and healthy fats like olive oil [104]. All of these components have been linked to a reduced risk of AF. This particular diet is useful both in the primary and secondary prevention of cardiovascular risk, including the reduction in oxidation stress. Fatty fish like salmon provide omega-3 fatty acids, which are beneficial for heart health. There is research showing that consuming lean fish, such as cod or saithe, lowers the risk of AF [105]. Patients who consume lean fish with a frequency of a minimum of three servings per week present a lower risk of AF than a group of never-consumers [103]. Studies have shown a U-shaped association between the consumption of marine fish and a lower risk of AF, providing greater benefits from a moderate intake of 0.63 g/day [106]. Additionally, extra virgin olive oil (EVOO) reduces the incidence of AF [107]. Olive oil is rich in monounsaturated fats, which can contribute to heart health by improving cholesterol levels. The effect of EVOO combined with the Mediterranean diet has shown a protective outcome for the new onset of AF [108]. The PREDIMED trial has proven to significantly reduce the risk of newly detected AF, with the goal of consuming 50 g or more per day of polyphenol-rich oil [109]. However, EVOO is also calorie-dense. Including it as part of a balanced diet, along with a variety of other Mediterranean diet elements such as fruits, vegetables, whole grains, and lean proteins, contributes to overall heart health.

Another dietary pattern reviewed for patients with AF is a plant-based diet (PBD). A PBD can have a positive impact on AF. The emphasis on a diet of vegetables and fruits may also have anti-inflammatory effects, potentially reducing the risk of AF [110]. A high fiber intake can support digestive health and improve cardiovascular function. PBD may contribute to maintaining a healthy weight, reducing the risk of obesity-related conditions that can lead to AF. On the other hand, lean meats, such as poultry and fish, provide essential nutrients like protein and omega-3 fatty acids. However, they can be replaced by plant-based sources of nutrients such as soy or nuts. In a six-month randomized clinical trial, the consumption of a range of cholesterol-lowering foods like nuts resulted in a significant 13% reduction of plasma LDL cholesterol [107]. Antioxidants in fruits and vegetables may help combat oxidative stress, which is linked to heart issues. Polyphenols, found in fruits (berries, apples, or grapes) and vegetables (spinach, kale, onions, and red cabbage), enhance the growth of Bacteroides, thereby decreasing the inflammatory burden [111]. Moreover, cruciferous vegetables (cabbage and broccoli) play a vital role in converting trimethylamine (TMA) to trimethylamine N-oxide (TMAO), which reduces ventricular remodeling and improves hemodynamic parameters, leading to a lower risk of AF [112]. Adopting a PBD can be a valuable part of a heart-healthy lifestyle.

A poor diet that includes fried, processed food in the context of AF can exacerbate risk factors associated with the condition. Ultra-processed foods should be excluded from the diets of patients with AF due to current European Society of Cardiology recommendations [107]. Fried food consumption has been associated with various risk factors for AF, such as inflammation or dyslipidemia [113]. Additionally, maintaining a healthy balance of electrolytes through diet may play a role in heart rhythm regulation. Studies have shown the tendency for an increased risk of AF in the lowest and highest daily salt intake in both women and men [114]. Not only fried foods but also low-carbohydrate diets enhance AF risk [108]. By making informed dietary choices, individuals with AF can positively influence their heart health and manage risk factors associated with the condition. It is an integral part of a comprehensive approach to living well with AF.

Limited clinical trials have been conducted on the effects of a ketogenic diet (KD) and intermittent fasting (IF) on AF [115]. KD is high in fat and low in carbohydrates. It causes the body to enter a state known as ketosis, where it mostly uses fats and ketones rather than carbohydrates for energy. It is suggested that KD might be considered for weight loss. Some studies suggest that KD may have potential benefits for managing AF. The diet’s impact on weight loss and insulin sensitivity could positively influence cardiovascular health, potentially reducing AF risk factors [115,116]. However, KD still requires careful attention and additional studies. Additionally, nutritional imbalances and limited fiber intake associated with KD may impact gut health, which has been linked to AF [117,118,119]. IF has gained popularity as a dietary approach involving cycles of eating and fasting. Zhang et al. evaluate the impacts of short-term IF on AF. IF could potentially aid in weight management, thus providing a possible lifestyle intervention to enhance the management of AF [120]. Some studies suggest that intermittent fasting may have potential benefits by decreasing blood pressure, insulin resistance, and oxidative stress [121,122,123].

However, more research is needed to fully understand the impact of various dietary patterns on AF and its long-term effects on cardiovascular health. Individuals considering diet modification, especially those with pre-existing heart conditions, should consult with healthcare professionals for personalized advice.

### 3.2. Coffee

The relationship between caffeine and AF is multifaceted. Caffeine, a methylxanthine contained in coffee, tea, cola, and energy drinks, has a neurohormonal and sympathetic nervous system effect [103]. While some studies have suggested a potential link between high caffeine intake and an increased risk of AF, others have not found a significant association. It is crucial to consider individual factors such as sensitivity to caffeine, health, and lifestyle.

A great number of studies are consistent in their statement of the neutral or even potentially protective effect of coffee on AF [124]. There is a possible protective mechanism for caffeine, the most active ingredient in coffee. The action of caffeine is antagonism of adenosine by competitive binding receptors, which reduces atrial muscle contractility and decreases sinoatrial node activity [125]. Moreover, polyphenols and caffeine found in coffee have antioxidant properties that may bind reactive oxidant species responsible for AF [126].

On the other hand, adenosine shortens the refractory period of atrial tissue and can trigger supraventricular arrhythmias [126]. Some research suggests that high doses of caffeine may have a more pronounced impact, especially in hypertensive patients, where AF is the most common arrhythmia [127]. An administration of very high doses, like 15 mg/kg of caffeine, resulted in sympathetic overreaction and pro-arrhythmic results [126].

However, individual responses may vary, and the protective effect, as well as the increase in AF risk from caffeine, is not definitely proven [125]. Although there is no clearly defined conclusion, a regular intake of up to 300 mg per day of caffeine appears to be safe [126]. It is essential to maintain a balanced approach and be mindful of individual sensitivity to caffeine.

### 3.3. Alcohol

The impact of alcohol on AF is multifactorial. Although there is little doubt that alcohol is independently associated with AF, its interactions with other risk factors are certain [128]. Ethanol may induce oxidative stress and the release of plasma fatty acids. Those indirect effects lead to AF, particularly in patients prone to cardiovascular diseases [129]. The direct effect is to shorten the atrial action potential, which leads to AF [128]. Undoubtedly, it is a crucial modifiable risk factor in the management of this tachyarrhythmia [130].

Excessive alcohol consumption has been linked to an increased risk of AF. Chronic alcohol intake can contribute to electrical disturbances and conditions such as hypertension and cardiomyopathy, which are risk factors for AF. Heavy alcohol intake triggers atrial activity, increases sympathetic tone, and reduces sodium channel expression. These effects provide for the maintenance of AF [130]. The studies showed a relative risk among people consuming high doses of alcohol (>6 doses/day) compared to those consuming <1 dose/day [129].

It is not only the amount but also the pattern of alcohol consumption that matters. Acute alcohol intake and the probability of AF are widely described with the term “holiday heart” [131]. Atrial-arrhythmia-related hospitalizations occurred more frequently in the period after holidays and weekends. This was inevitably connected with higher alcohol ingestion during vacation and free time.

On the contrary, moderate alcohol consumption, <15 g/day for women and <30 g/day for men for ethanol, has a protective effect in coronary cerebrovascular and peripheral vascular artery diseases and in metabolic syndrome [129]. Particularly, red wine, part of the Mediterranean diet, has been associated with certain cardiovascular benefits. Resveratrol, a compound found in red wine, has antioxidant and vascular properties [103].

However, even moderate alcohol intake could potentially lead to AF [103]. Undoubtedly, it contributes to many risk factors, such as hypertension and obesity [128]. Otherwise, a small amount of alcohol is considered cardioprotective, and every patient should be treated individually.

## 4. New-Onset AF Risk Assessment Based on Lifestyle

Researchers created various risk scores to predict the risk of AF. The Framingham AF risk score created by Schnabel et al. includes age, weight, blood pressure, heart failure, and ECG results [132]. The ARIC risk model counts the following: smoking, age, height, race, comorbidities such as diabetes or HF, ECG, and echocardiography results [133]. The CHARGE-AF score also included age, height, race, blood pressure, comorbidities such as diabetes, previous heart attack, and HF [133].

In 2023, Segan et al. created the HARMS_2_-AF lifestyle risk score, which allows the easy assessment of the risk of AF based on lifestyle and identifies people at increased risk. Based on the study of 314,280 people during an average observation period of 12.9 years, AF occurred in 5.7%. These patients were mainly elderly men (IQR 59–66 years), with arterial hypertension (67.2%), smokers (57%), and moderately high alcohol consumption [median 13.5 (IQR 6.0–25.5) standard drinks per week; 1 standard drink = 8 g of alcohol]. The BMI of patients with AF was, on average, 27.9 kg/m^2^, of whom 33.2% were obese. The research showed that these variables and the presence of sleep apnea are statistically significant predictors of AF (*p* < 0.001). The HARMS_2_-AF indicator is based on a scale of 0–14 points and counts the following factors: [H] arterial hypertension (4 points), [A] age (60–64 years: 1 point; ≥65 years: 2 points), [R] raised BMI ≥ 30 kg/m^2^ (1 point), [M] male sex (2 points), [S] sleep apnea (2 points), smoking (1 point), and [AF] alcohol (7–14 standard drinks/week: 1 point; ≥15 standard drinks/week: 2 points) (Table 3) The research showed a linear dependence between the HARMS_2_-AF score and the occurrence of AF. Result ≥ 5 points is associated with an increased risk of AF (5–9 points: HR 12.79; 10–14 points: HR 38.70), and it is an indication for major changes in lifestyle [134]. 

The risk scores described in this chapter are composed of different variables and constants. They also have differential predictive performance. For respective indicators: Framingham AF risk score: AUC 0.568; ARIC risk model: AUC 0.713; CHARGE-AF score: AUC 0.754; HARMS2-AF score: AUC 0.757 [132,133,134]. The HARMS_2_-AF score has not only the highest predictive performance but also easy-to-assess risk factors that do not require tests like echocardiography or electrocardiography. The assessment with this scale is possible in every consulting office, in the patient’s home, or at the bedside. Moreover, this AF risk score does not generate any additional costs. This score may become a method of AF population screening. Furthermore, many variables included in the HARMS_2_-AF score, such as weight, smoking, or alcohol consumption, are modifiable. It gives the opportunity to implement changes in patients’ lifestyles in order to reduce the risk of AF in the future [134].

## 5. Conclusions

With a current prevalence of 2% among people younger than 65 years old and about 9% among people ages 65 and older, AF is a very significant disease nowadays. In this review, we focused on several factors that may have an impact on AF development and management. We paid particular attention to lifestyle, including physical activity, smoking, the impact of sleep disorders, air pollution, and the broad aspect of diet. Literature data indicate an unquestionable association between the factors we presented and the occurrence and treatment of AF. Engaging in physical activity proves to be a proactive measure for reducing AF incidence and limiting associated complications. We also highlighted the pathomechanism of AF during tobacco smoking. Both smoking traditional cigarettes and non-combustible tobacco products are crucial risk factors for AF, with the increasing significance of passive smoking in AF pathogenesis. Exploring the processes occurring in the body during sleep disorders such as AS and OSA, we found that they are not only a risk factor for arrhythmia development but also induce a poorer response to antiarrhythmic therapy. Air pollution is also a significant factor in AF development. Regardless of the duration of exposure to air pollutants, analyses have clearly shown an increased incidence of AF among exposed individuals. Analyzing the dietary aspect in the context of AF, it turns out that each diet has its own set of advantages and disadvantages. Therefore, to optimize well-being, patients should be provided with the information and tools necessary for adhering to a diet tailored to their specific conditions and the degree of AF advancement, with particular attention to excluding ultra-processed foods, following the recommendations of the European Society of Cardiology.

Consequently, early detection of these factors and prompt intervention should be an integral part of clinical practice. Considering changes in diet and lifestyle as preventive measures for AF and complementary to existing arrhythmia treatment methods is essential. Integrated treatment strategies, encompassing all relevant aspects, provide the most comprehensive solution for patients dealing with AF.

## Figures and Tables

**Table 1:** Types of training used in patients with AF.

Type of Training	Paper	Training Program	Duration and Frequency	AF Type
MICT	Reed et al. (2018) [37]	(1) 10–15 min aerobic warm-up; (2) 30 min continuous aerobic training (e.g., elliptical trainers, jogging, walking, and cycling) with 67% to 95% of peak heart rate; and (3) 15 min strengthening and stretching exercises	60 min twice a week	Persistent or permanent
HIIT	Reed et al. (2018) [37]	(1) a 2 min warm-up at 50% of peak power output; (2) two 8 min interval training blocks of 30 s work periods at 80%to 100% of peak power output interspersed with 30 s active recovery (16 min conditioning phase), and 4 min of recovery between the blocks; and (3) a 1 min cooldown at 25% of peak power output on an upright cycle ergometer.	23 min twice a week	Persistent or permanent
AIT	Malmo et al. (2016) [39]	walking or running on a treadmill: (1) 10 min warm-up at 60% to 70% of peak heart rate, (2) four 4 min intervals at 85% to 95% of HR peak with 3 min of active recovery at 60% to 70% of HR peak between intervals, and (3) 5 min cooldown	43 min 3 times a week	Paroxysmal or persistent

Abbreviations: MICT—Moderate-to-vigorous intensity continuous training, HIIT—High-intensity interval training, AIT—Aerobic interval training, and AF—Atrial fibrillation.

**Table 2:** Outcomes of rehabilitation with various training methods [37,39].

	MICT		HIIT		AIT	
	Baseline	Change	Baseline	Change	Baseline	Change
Physical functioning ^a^	43.8 (8.8)	2.7 (6.2)	41.8 (9.2)	1.9 (5.8)	50.6 (6.7)	1.2 (3.9)
Bodily pain ^a^	37.3 (7.3)	−1.5 (8.5)	38.5 (7.0)	−1.5 (9.0)	53.1 (9.2)	1.3 (6.8)
General health ^a^	42.5 (10.0)	1.4 (7.2)	44.5 (9.2)	0.7 (6.1)	49.0 (8.7)	4.4 (7.0)
Vitality ^a^	45.6 (9.3)	3.1 (8.7)	45.6 (10.5)	4.4 (10.2)	47.1 (9.8)	8.2 (8.2)
Social functioning ^a^	49.4 (9.6)	0.9 (6.7)	49.2 (10.4)	2.2 (6.1)	50.9 (9.4)	0.9 (12.0)
Mental health ^a^	52.4 (7.6)	−0.4 (6.1)	51.8 (8.7)	2.4 (8.1)	52.5 (6.8)	3.0 (6.9)
Physical component score ^a^	37.9 (8.8)	1.1 (4.9)	38.4 (7.1)	0.5 (6.1)	50.3 (8.8)	2.2 (4.4)
Mental component score ^a^	53.4 (10.7)	−0.2 (7.6)	53.0 (10.3)	2.8 (8.4)	50.6 (8.4)	3.6 (6.5)
Time in AF, % ^b^	98.1 (4.8)	−6.2 (23.2)	93.8 (15.7)	0.1 (0.5)	8.1 (11.2)	−3.3 (7.2)
Body mass index ^c^	29.9 (6.2)	−0.3 (0.9)	30.9 (5.7)	−0.1 (1.0)	28.2 (4.8)	−0.5 (0.9)
Systolic blood pressure, mm Hg	127.5 (15.6)	−1.8 (11.8)	123.8 (18.3)	1.1 (14.9)	135.0 (11.0)	−3.0 (10.3)
Diastolic blood pressure, mm Hg	79.6 (9.4)	−2.7 (7.0)	77.2 (11.2)	−0.3 (11.2)	81.0 (8.0)	−2.0 (7.0)

^a^ Short Form-36 Health Survey (SF-36); ^b^ measured with 24 h Holter monitor; ^c^ calculated as weight in kilograms divided by height in meters squared; and SD in brackets. Abbreviations: MICT—Moderate-to-vigorous intensity continuous training, HIIT—High-intensity interval training, AIT—Aerobic interval training, and AF—Atrial fibrillation.

**Table 3:** HARMS2-AF lifestyle risk score [134].

		Points (0–14)
H	Hypertension	4
A	Age	
	(60–64 years)	1
	(≥65 years)	2
R	Raised BMI (≥30 kg/m^2^)	1
M	Male sex	2
S	Sleep apnea	2
S	Smoking	1
AF	Alcohol	
	(7–14 standard drinks/week)	1
	(≥15 standard drinks/week)	2

## Data Availability

The data used in this article are sourced from materials mentioned in the References section.
